# Application of magnifying narrow-band imaging endoscopy for diagnosis of early gastric cancer and precancerous lesion

**DOI:** 10.1186/1471-230X-11-135

**Published:** 2011-12-14

**Authors:** Jing Zhang, Shi-Bin Guo, Zhi-Jun Duan

**Affiliations:** 1 Department of Gastroenterology, the First Affiliated Hospital of Dalian Medical University, Dalian 0086-116011, Liaoning Province, China; 2 This auther is currently working in Dalian Centric Hospital, Dalian 0086-116001, Liaoning Province, China

## Abstract

**Background:**

Gastric carcinoma is the second commonest cause of cancer deaths worldwide. Early detection and diagnosis of gastric cancer in the stomach is important for improving the prognosis of gastric cancer. This retrospective study was designed to investigate the value of magnifying narrow-band imaging (NBI) in the diagnosis of precancerous lesions and early gastric cancer.

**Methods:**

This study included 122 patients who were diagnosed with early gastric cancer or precancerous gastric lesions by endoscopy. The patients underwent an examination with conventional endoscopy, magnifying NBI, and magnifying chromoendoscopy. Images resolution was evaluated, and the morphology, pit patterns and blood capillary forms of lesions were analyzed. The presence of gastric carcinoma and high grade intraepithelial neoplasia in the biopsy samples was considered as a positive pathological result, which is used to assess accuracy of endoscopic diagnosis.

**Results:**

For image resolution, magnifying NBI and magnifying chromoendoscopy were significantly superior to magnifying conventional endoscopy in morphology, pit pattern and blood capillary form (P < 0.01), and magnifying NBI was significantly superior to magnifying chromoendoscopy in blood capillary form (P < 0.01). IV, V_1_, and VI type of gastric pit pattern were detected in 14 cases, 43 cases, and 17 cases in patients with high grade intraepithelial neoplasia, respectively. V_1 _and VI type of gastric pit pattern were detected in 9 cases and 39 cases in patients with early gastric cancer, respectively. The presence of irregular minute vessels and variation in the caliber of vessels was found in 109 cases. The accuracy, sensitivity, specificity, false positive rate and false negative rate for diagnosis of early gastric cancer and precancerous gastric lesions were 68.9%, 95.1%, 63.1%, 24.5%, and 32.4% for conventional endoscopy, 93.6%, 92.7%, 94.5%, 5.7%, and 6.9% for magnifying NBI, and 91.3%, 88.6%, 93.2%, 13.2%, and 21.48% for magnifying chromoendoscopy, respectively.

**Conclusions:**

This study demonstrates that magnifying NBI is superior to conventional endoscopy in the diagnosis of early gastric cancer and precancerous gastric lesions, and can be used for screening early malignancies of the stomach.

## Background

Gastric carcinoma is one of the most common malignant tumors, and is the second commonest cause of cancer deaths worldwide [1]. About 870000 new gastric cancer cases are diagnosed every year on a global scale. There is a considerable geographical variation in the incidence of this cancer [2]. It is reported that incidence rates are high in eastern Asia, and low in Europe, North America and Africa [1]. Chinese have high gastric cancer rates with about 30% new cases diagnosed each year, which are only less than those found in Korea and Japan [3]. The prognosis of gastric carcinoma is closely related to the stage of disease at the time of diagnosis. The survival rate is greater than 90% in 5 years [4] for early gastric cancer, but the prognosis is very poor for advanced gastric cancer. Therefore, early diagnosis of gastric cancer is very important for an excellent prognosis. However, since most patients with early gastric carcinoma do not have specific symptoms, it is difficult to distinguish early gastric carcinoma from benign peptic ulcer or gastritis, and only about 10-20% of gastric cancers are diagnosed as early cancers in many countries [3]. With the development of technology, some endoscopic imaging modalities, such as magnifying narrow-band imaging (NBI) endoscopy, have been used recently for the diagnosis of early gastric cancer. Although many studies have reported that this endoscopic imaging modality can increase the rate of diagnosis of early cancers and precancerous lesions by enhancing visualization [5,6], there are few studies about comparison between NBI and conventional endoscopy. This retrospective study was designed to compare the accuracy and sensitivity of magnifying NBI endoscopy with conventional endoscopy and chromoendoscopy for diagnosis of precancerous lesions and early gastric cancer.

## Methods

### Patients

This retrospective study was performed in the Department of Gastroenterology, Dalian Central hospital. During January 2008 to January 2011, a total of 14389 patients underwent endoscopic examination, and 526 lesions were detected. Among them, 122 patients were diagnosed as early gastric cancer or precancerous gastric lesions by the pathologic results, and were enrolled in this retrospective study. The ages of the122 patients (83 males and 39 females) ranged from 16 to 94 years with a mean age of 63.5 ± 14.1 years. Early gastric cancer was defined as cancer confined to the mucosa or submucosa regardless of lymph node metastasis. In our study, precancerous lesions referred to high grade intraepithelial neoplasia, which included intraepithelial carcinoma and severe dysplasia according to the Vienna classification of gastrointestinal epithelial neoplasms [7]. All the lesions were confirmed by pathologic diagnosis of endoscopic resection or post-surgery tissue. Exclusion criteria were pre-existing or advanced gastric cancer, recent upper gastrointestinal bleeding or coagulation disorders, and severe comorbidities that may affect tolerance to upper endoscopy. The presenting manifestations were abdominal pain in 88 cases (72.1%), distension in 52 cases (42.6%), heartburn in 60 cases (49.2%), belching in 28 cases (23.0%), acid regurgitation in 32 cases (26.2%), and nausea in 29 cases (23.8%).

### Procedures

**Figure 1:**
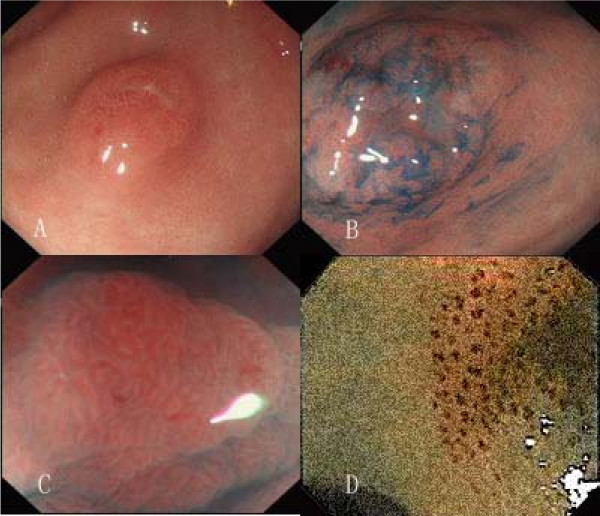
**Demonstration of gastric precancerous lesion with conventional endoscopy(A) and iodine staining pattern (B); Pit pattern of this precancerous lesion with magnifying chromoendoscopy (C) and Blood capillary form with magnifying NBI (D)**.

The patients were first given careful observation in order to identify any abnormalities of the surface or the color with conventional endoscopy. After detecting lesions, magnifying chromoendoscopy and NBI magnifying endoscopy examination were followed on the same day or on another day. The morphology, pit pattern and blood capillary form of lesions were observed and recorded to determine that the lesion was malignant or benign (Figure 1). Then, biopsies were taken and studied by the same expert pathologist. The image quality was scored by two other endoscopists during the examination. Pathological diagnosis was regarded as the gold standard, and was used to assess accuracy of endoscopic diagnosis. Once lesions were diagnosed as gastric cancer or precancerous lesions, patients were underwent endoscopic resection or surgery. This study was approved by the Medical Science's ethics committee of Dalian Central hospital, patients were informed of the possible risks and benefits of participation in the study, and written informed consent was obtained from all the patients or their relatives before their examination.

### Instruments

The NBI main unit was an Olympus CV-260SL and the endoscope was an Olympus GIF-H260Z (80×) (Olympus Corporation, Japan); 1.2% iodine was used for staining (Micro-Tech, Nanjing, CO., LTD).

### Image evaluation

The image quality was scored as follow: 1 point: obscure; 2 points: clouding; 3 points: more clear; and 4 points: clear [8].

### Statistical analysis

Data analysis was performed using SPSS 10.0 software (Chicago, IL, USA). Analysis of variance (ANOVA) or Wilcoxon statistical methods were used to determine statistical significance. All measurements in this study were expressed as mean ± SD. P < 0.05 was considered statistically significant.

## Results

**Figure 2:**
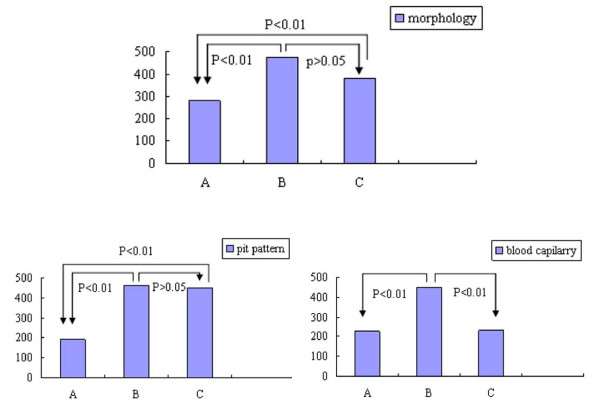
**Comparition of image quality about morphology, pit pattern and blood capillary form among conventional endoscopy, magnifying NBI and magnifying chromoendoscopy**. A represent conventional endoscopy; B represent NBI; C represent chromoendoscopy.

A total of 122 cases were diagnosed as early gastric cancer or precancerous lesions by pathologic diagnosis of endoscopic resection or post-surgery tissue. The lesions were located in gastric antrum in 67 cases (54.9%), gastric angle in 29 cases (23.8%), gastric body in 22 cases (18.0%), and cardia and gastric fundus in 4 cases (3.3%). The image quality of 3 modes was compared concerning the morphology, pit pattern and blood capillary form of early gastric cancer and precancerous lesions. We found that magnifying NBI and magnifying chromoendoscopy were significantly superior to magnifying conventional endoscopy in respect of morphology, pit pattern and blood capillary form (P < 0.01), and magnifying NBI is significantly superior to magnifying chromoendoscopy in respect of blood capillary form (P < 0.01). There is no significant difference between magnifying NBI and magnifying chromoendoscopy with regard to morphology and pit pattern (P > 0.05) (Table 1, Figure 2).

**Table 1:** Comparison of image quality among three endoscopic modalities

*item*	*mode*	*Score of image quality*				*total score*
			
		1	2	3	4	
	conventional endoscopy	26	33	63	0	281
morphology	magnifying NBI	0	0	12	110	476
	magnifying chromoendoscopy	8	19	45	50	381
	conventional endoscopy	26	62	34	0	190
pit pattern	magnifying NBI	0	0	25	97	463
	magnifying chromoendoscopy	0	0	40	82	448
	conventional endoscopy	40	59	23	0	227
blood capillary form	magnifying NBI	0	0	37	85	451
	magnifying chromoendoscopy	38	57	27	0	233

The gastric pit pattern is classified into six types according to Sakaki [9]. In our studies, a total of 122 patients with early gastric cancer or precancerous gastric lesions were diagnosed, including 74 cases of high grade intraepithelial neoplasia and 48 cases of early gastric cancer. In patients with high grade intraepithelial neoplasia, IV type of gastric pit pattern was detected in 14 cases, V_1 _type of gastric pit pattern was detected in 43 cases, and VI type of gastric pit pattern was detected in 17 cases; while in patients with early gastric cancer, V_1 _type of gastric pit pattern was detected in 9 cases, and VI type of gastric pit pattern was detected in 39 cases. We also observed blood capillary form of gastric lesions. The disappearance of the normal mucosa capillary network or the presence of microvessels with irregular size, shape and distribution were found in 109 cases.

The accuracy, sensitivity, specificity, false positive rate and false negative rate of conventional endoscopic diagnosis for early gastric cancer and precancerous gastric lesions were 68.9%, 95.1%, 63.1%, 24.5% and 32.4%, respectively, while the corresponding values of magnifying NBI were 93.6%, 92.7%, 94.5%, 5.7% and 6.9%, respectively, and the corresponding values of magnifying chromoendoscopy were 91.3%, 88.6%, 93.2%, 13.2% and 21.48%, respectively. Compared with conventional endoscopic, magnifying NBI was superior in the diagnosis of early gastric cancer and precancerous gastric lesions. However, there was no diagnostic difference between magnifying NBI and magnifying chromoendoscopy.

## Discussion

Gastric cancer is one of the main causes of cancer death in China. Most of these patients have poor prognosis because of late presentation and diagnosis. The best tactics for dealing with gastric cancer is prevention, early detection and early treatment. It is generally believed that gastric cancer is a multi-step progression from chronic gastritis to gastric atrophy, intestinal metaplasia, dysplasia and cancer. Early gastric cancer is defined as a cancer confined to the mucosa or submucosa regardless of lymph node metastasis [10]. If gastric cancer can be detected in early stage, the prognosis for gastric cancer is excellent, the survival rate is greater than 90% in 5 years, and curative endoscopic resection may be possible in some cases with early gastric cancers and precancerous gastric lesions, without the need for surgery. Therefore, it is necessary to mass screen for symptomatic groups in high-incidence areas, and endoscopy has been considered as one of the most useful tool for detecting early gastric cancer. However, at present, only about 4-10% of patients with gastric cancer are diagnosed as early cancer in China, and the missed diagnosis of gastric cancer on endoscopy is a common occurrence. It is reported that the false-negative rates are high up to 5-19% [11,12]. There may be two reasons for this situation. One reason is that the symptoms of early gastric cancer are not specific to distinguish from those of gastritis and benign peptic ulcer disease. In our group, about 90% of patients with early gastric carcinoma have heartburn, abdominal pain and discomfort in the upper abdomen. The other reason is that some of the lesions are so subtle that they are overlooked by inexperienced endoscopist. Without being aware of early gastric cancer, they pay much attention to the detection of gross lesions, rather than tiny changes in color, vascularity or texture, which are distinctive characteristics of early gastric cancer. There has been great advancement about the technology of endoscopic imaging in recent years, and these new technologies have improved the sensitivity in identifying early gastric cancer.

Magnifying endoscope (GIF-Q240Z) used in our study can provide magnified images up to x80. This new magnifying endoscope can detect minute changes of gastric mucosal surface, such as the color of lesions (same color as surrounding tissues, red or pale), the surface of lesions (flat, elevated or depressed), the presence of granules or nodules, with or without ulcer and fold change. Irregular and destruction of the minute surface pattern and color change of mucosal surface were all considered as characteristics of precancerous gastric lesion or early gastric cancer [13-15].

Dye spray chromoendoscopy can enhance the recognition of minute structural alteration caused by neoplastic changes, which is difficult to be perceived by conventional endoscopy. Magnifying chromoendoscopy is also useful in observing the surface mucosal pattern and capillary structure. By analyzing the surface structure pattern, histological changes of carcinoma, dysplasia, adenoma and hyperplasia might be suspected. Since histopathological examination of biopsy material is very important for the final diagnosis, accurate biopsy contributes to acquire the correct diagnosis of the lesion. Magnifying chromoendoscopy can improve the diagnosis of early gastric cancer and precancerous lesions in the stomach by facilitating the identification and biopsy of abnormal areas. It is difficult in some cases to identify the margins of the lesions by conventional endoscopy, especially those of superficial or flat-type lesions. Magnifying chromoendoscopy has an advantage in coping with this difficulty [13,16].

Narrow-band imaging (NBI) is a new kind of endoscopic technology designed to enhance the contrast of the mucous membrane without staining. NBI uses special narrow-band filters which filter broad-band spectrum to leave a narrow-band spectrum for the diagnosis of digestive tract disease [17-19]. NBI endoscopy significantly improves diagnostic accuracy in two ways. First, because the shorter wavelength light left by the special narrow-band filter can not penetrate deeply into the mucosa, NBI improves visibility of mucosal pit pattern. Second, since the specific wavelength 415 nm left by special narrow-band filter corresponds to the peak absorption spectrum for hemoglobin [20], magnifying the image with NBI can give important information about microvascular pattern [21].

In our study, we find that the image quality of magnifying NBI is superior not only to magnifying conventional endoscopy in respect of morphology, pit pattern and blood capillary form of abnormal areas, but also to magnifying chromoendoscopy concerning blood capillary form. However, in our study, the image quality was scored during the examination, and the scoring system itself has subjective nature, so it would be better to score the image quality in a blinded fashion by more investigators. We also find that the diagnotic accuracy of early gastric cancer and precancerous gastric lesions by magnifying NBI is significantly higher than that of conventional endoscopy. Moreover, NBI has all the functions that conventional endoscopy has, and the NBI and conventional endoscopy pattern can be easily switched just by pushing one button [22,23].

Both NBI and chromoendoscopy can show the enhanced mucosal pattern and the microvascular structure of the mucosa by the amplificatory function[13], and mucosal pattern and microvascular structure have been regarded as distinctive characteristics of early gastric cancer and precancerous gastric lesions.

It has been known that angiogenesis is an important factor in gastrointestinal carcinogenesis [24], suggesting that the vascular pattern of gastric cancer and precancerous gastric lesion is differ from that of normal mucosa [25]. Therefore, observation of vascular pattern contributes to diagnosis of such lesions [26]. Nakayoshi [5] et al observed the microvascular networks of 165 patients with early gastric carcinoma with magnifying NBI, and found that 66.1% of differentiated adenocarcinoma had fine microvascular networks, and 85.7% of undifferentiated adenocarcinoma had corkscrew microvascular networks. Liu [27] et al also drawn the conclusion that vascular architecture was helpful in the identification of early gastric cancer. They studied the microvascular architecture with confocal endomicroscopy, and demonstrated that differentiated gastric cancerous mucosa showed hypervascularity and various caliber microvessels with irregular shapes, and undifferentiated gastric cancer showed hypovascularity and irregular short branched vessels. In our study, we found abnormal capillary patterns in 109 cases including disappearance of the normal mucosa capillary network, tortuous microvessels with irregular length and irregular arrangement, and variation in the caliber of vessels or even dilated microvessels with tortile tips. However, at present, as a new technology, there is no unified standard for diagnosis of early gastric carcinoma by using microvascular architecture.

Many studies have shown that gastric mucosa patterns of gastric cancer and precancerous gastric lesions are characteristic. Tanaka et al classified pit patterns of gastric mucosa into five types, and pointed out that differentiated tubular adenocarcinomas mainly showed the type IV, and poorly differentiated tubular adenocarcinomas mainly showed type V [17]. Yoshida et al distinguished gastric cancer and precancerous gastric lesions from controls by analyzing the surface structure pattern [28]. In our study, V_1 _and VI type of gastric pit pattern are the most common mucosa pattern of early gastric cancer and precancerous lesions. Therefore determination of pit pattern as well as microvascular architecture is very important for detecting early gastric cancer and precancerous lesion [29].

## Conclusions

Magnifying NBI endoscopy has advantages in the detection of some lesions with minute changes of gastric mucosa pattern and capillary form, and facilitates the diagnosis of early gastric cancer and precancerous gastric lesions by identification and biopsy of the lesions. Clearly, it can be used for the screening of early malignancies of the stomach.

However, since the present study is uncontrolled, and the number of cases is not big enough, a further prospective, blinded study with a sufficient number of patients who have or have no gastric carcinoma is needed to assess the value of magnifying narrow-band imaging in the diagnosis of precancerous lesions and early gastric cancer in clinical practice.

## Competing interests

The authors declare that they have no competing interests.

## Authors' contributions

JZ and ZJD designed the experiment, JZ and SBG performed the experiments, analyzed the data, SBG and JZ wrote the manuscript, ZJD revised it. All authors read and approved the final manuscript.

## Pre-publication history

The pre-publication history for this paper can be accessed here:

http://www.biomedcentral.com/1471-230X/11/135/prepub
